# Attitude of pregnant women towards Normal delivery and factors driving use of caesarian section in Iran (2016)

**DOI:** 10.1186/s13030-019-0149-0

**Published:** 2019-04-01

**Authors:** Soraya Siabani, Khadijeh Jamshidi, Mohammad Mehdi Mohammadi

**Affiliations:** 1 School of Public Health, Kermanshah University of Medical Sciences and Registered External Supervisor at the University of Technology Sydney (UTS), Kermanshah, Iran; 2 School of Public Health, Kermanshah University of Medical Sciences, Kermanshah, Iran; 3 School of Nursing and Midwifery, Kermanshah University of Medical Sciences, Kermanshah, Iran

**Keywords:** Pregnant women attitude, Vaginal delivery, Cesarean section, Pregnancy, Iran

## Abstract

**Background:**

Normal delivery is a natural and physiological process with numerous benefits for mother and baby. Giving birth by Caesarean Section (CS) should be limited to the cases in which normal delivery is not possible. The purpose of the study was to determine the attitudes of pregnant women towards Normal Delivery and factors driving the use of Caesarian Section in Kermanshah, Iran.

**Methods:**

This analytical-descriptive study was conducted on 410 pregnant women referred to the PHC centers in Kermanshah in western Iran. They had been selected through a multi-stage sampling method, including clustering, randomized, and proportional sampling, from among all eligible women. Data was collected using a questionnaire standardized by previous studies. The level of 0.05 was considered significance association, whenever applied.

**Results:**

The mean and standard deviation for participant age was 27.65 ± 5.37 years. The median score for participant attitude was 60.7 ± 9.5 (range from 22 to 85). Generally, 21.5% had a negative attitude toward normal delivery and preferred CS. Participant attitude was negatively correlated with a pregnant woman’s age, lower age, and a more positive attitude towards vaginal childbirth. The attitude of women with a history of normal delivery was 63 ± 9 and for those with a history of CS was 56.7 ± 9.3, significantly different.

**Conclusion:**

Most women had a positive attitude towards normal delivery, particularly those who had experienced normal delivery in their previous childbirth. Although only a quarter of the participants had a negative attitude toward normal delivery, this figure still was of utmost significance, therefore educational interventions, specifically encouraging women with history of normal delivery to consult their peers, are recommended.

## Background

The advantages of normal delivery (vaginal delivery) for both mother and baby have been reported in numerous studies [1–4]. Childbirth through abdominal surgery, called Cesarean Section (CS), has been done for millions of mothers and babies over the past centuries. However it should be limited to the cases in which vaginal childbirth is not possible or normal delivery is subject to serious risks for the baby or mother [1]. Numerous complications may arise for mothers and babies due to CS, including the general surgical complications (e.g. fever, infections, bleeding, scarring, long time bed restand complications of anesthesia), and many specific complications such as urinary tract involvement, hysterectomy, child-mother relationship issues etc. [3, 4]. Also, while the mortality rate for elective CS has been reported to be about 6 in 100,000 cases, the rate for vaginal childbirth is 2 in 100,000 cases [5]. Cesarean section is used frequently in both developed and developing countries, especially in Asia (more than 50% of childbirths in China) [6]. In Iran, similarly, the CS rate is much higher than the standard rate (5 to 15%) that is expected by the World Health Organization [7]. The results of a study conducted in Tehran (Iran) showed that 66% of deliveries had been performed through CS [8]. The factors contributing CS in Iran can be divided into two main categories; those related to mother and those related to medicine/doctors. In general, medical issues, such as maternal age, being the first childbirth, history of CS, being a candidate for tubal ligation, and fear of painful vaginal childbirth, have been listed as contributing factors [9]. Nowadays, socioeconomic issues and financial issues play a significant role in the choice of CS by physicians who encourage women to choose CS [10, 11]. Further, the negative attitudes might be due to incorrect beliefs of people about health related problems, either about the baby or mother. Other less common reasons may be following vogue and fashion or disrespect by the medical staff of hospitals prior to the processes of natural childbirth. Also, concern about increasing the risks for the baby in normal delivery being traumatized about the vaginal area leading to sexual dysfunction have been reported as the most important incentives for mothers to select CS [12].

However, there is little information about women attitudes in western Iran, especially in the city of Kermanshah, about CS childbirth. People beliefs and attitudes are important determinants for their behavioral modification, therefore, before any health related intervention program, knowing their attitudes towards the issue can play an important role in adopting strategies and making decisions by healthcare organizations in order to reduce the rates of cesarean section delivery. Thus, the present study was conducted to determine the attitudes of pregnant women towards Normal Delivery and factors driving use of Caesarian Section in Kermanshah, Iran.

## Materials and methods

This descriptive analytical, cross-sectional study, approved by the ethics committee at Kermanshah University of Medical Sciences (KUMS), was conducted in 2016 in nine primary healthcare (PHC) centers located in Kermanshah in western Iran. The study population was pregnant women who were provided with primary health care by the PHC centers. The necessary sample size was calculated at 375 individuals using the following formula and applying means and standard deviation from similar previous studies. Considering 10% of non-response, 410 eligible pregnant women were selected via a multi-stage sampling method that included random sampling (selection of healthcare centers), quota sampling (selection of the number of participants from each healthcare center), and then simple random sampling to select each quota as the study participants.

$$ n=\frac{Z^2p\left(1-p\right)}{d^2} $$

The quota for PHC centers in terms of the number of pregnant women covered in each center was as follows: 25 women (6.1%) selected from Haj Daei Healthcare, 15 women (3.7%) from Samimi Healthcare, 63 women (15.4%) from Keihanshahr Healthcare, 42 women (10.2%) from Moalem Healthcare, 62 women (15.1%) from Farhangian Phase 2 Healthcare, 63 women (15.4%) from Pardis Healthcare, 89 women (21.7%) from Shahid Rajaei Healthcare, 20 women (4.9%) from Sina Healthcare, and 31 women (7.6%) from Kashani Healthcare.

The data collection instrument for the measurement of attitudes was a questionnaire borrowed from another study [13], but with some modification. After modification, its face validity was determined through a pilot study and by obtaining the opinions of experts and professors in the School of Midwifery. During this process, minor changes were made in the appearance of some of the items in the given questionnaire. A few new items (items 5, 6, 7, 8, 9 and 15) were added to the original questionnaire. The reliability of the questionnaire was similarly measured through Cronbach’s alpha coefficient, which was confirmed by 80%. The questionnaire consisted of two parts; in its first part, demographic information (age, occupation, level of education, type of health insurance, and place of living) were collected. The second part of the questionnaire comprised items examining the attitudes of pregnant women towards natural childbirth. In terms of ranking the items, a 4-point Likert-type scale was used. A quite negative attitude to natural childbirth was assigned a score of 1 and a quite positive attitude to natural childbirth was assigned a score of 4 (the scores for questions 14 through 20 are in reverse). The total score of the questionnaire is the result of summing the points for each item, so the score range for attitude was from 22 (the lowest attitude to natural delivery) to 88 (the highest attitude to natural delivery). In order to ease the interpretation of the results in this study, scores 22–54 were considered negative attitudes and scores 55–88 positive. The data collection was completed by two health care nursing experts who interviewed the participants following a full explanation of the study and after obtaining informed consent forms. The data were analyzed using the SPSS version 16. To describe the findings, descriptive statistics (frequency, mean, and standard deviation) and to test the relationships, analytical statistics (t-test, Pearson correlation coefficient, Analysis of Variance (ANOVA), and Chi-square test) were employed at the 0.05 level of significance.

## Results

The mean and standard deviation for participant age (μ ± σ) was 27.65 ± 5.37 years. The mean age for their husbands was 32.31 ± 5.77 years. As well, 89.8% of these women were housewives and 25.4% of the husbands were self-employed. In terms of level of education, 28.5% of the pregnant women and 34.4% of their husbands had a diploma (Table 1). Moreover; 39.5% of the households were covered by social security insurance, 18.3% benefited from healthcare service insurance, 7.6% had military staff insurance, 1.2% were covered by Rostaei insurance (a type of public health care insurance providing for those living in rural areas of Iran), 0.2% used Imam Khomeini Relief Committee insurance, 1.2% had no insurance, and 32% were covered by other insurances.

**Table 1:** Frequency distribution of the variables of occupation and level of education among pregnant women participating in the study and those of their husbands

	Pregnant women	Husbands
	Frequency (percentage)	Frequency (percentage)
Occupation		
Employee	28 (6.8)	104 (25.4)
Worker	3 (0.7)	92 (22.4)
Self-employed	–	98 (23.9)
Retired	–	1 (0.2)
Unemployed	–	9 (2.2)
Housewife	368 (89.8)	–
Other	11 (2.7)	106 (25.9)
Level of education		
Illiterate	14 (3.4)	5 (1.2)
Primary school	57 (13.9)	34 (8.3)
Middle school	103 (25.1)	89 (21.7)
Diploma	117 (28.5)	141 (34.4)
Associate’s degree	39 (9.5)	43 (10.5)
Bachelor’s degree and higher education	80 (19.5)	98 (23.9)

The results revealed that 46.1% of the pregnant women had no history of previous childbirth (the current pregnancy was their first), 36.1% of these women had had a previous delivery, 16.1% of them had experienced two or three deliveries, and 1% had a history of more than three deliveries. The mean for the number of children was 1.39; in this respect, 47% of women had no children, 36.3% had just one child, 12.9% of them had two children, and the rest had more than two children. Furthermore, the delivery mode among women who had a history of previous childbirth was natural in 58.8% of women and CS in 41.2% of women. As well, 17.8% of the pregnant women were in the first trimester of pregnancy, 43.2% of them were in the second trimester, and 39% were in the third trimester.

The mean and standard deviation for the attitudes was (μ ± σ) 60.7 ± 9.5 with a range between 22 and 85. Overall, 78.5% of the women had a positive attitude and 21.5% had a negative attitude toward natural childbirth.

Attitudes were inversely correlated with a pregnant woman’s age in a way that the younger the age the more positive the attitude towards natural childbirth (r = − 0.1, *p* = 0.02). Attitudes were also inversely correlated with the age of the pregnant woman’s husbands, but the given correlation was not statistically significant (r = − 0.06, *p*>0.05). There was a positive correlation between the attitude and the age at pregnancy, but this correlation was not significant (r = 0.06, p>0.05).

The mean attitude of women with a history of childbirth was 60.9 ± 9.5 and the value for women with a history of previous CS delivery was 60.4 ± 9.6, the difference was not statistically significant (*p*>0.05). The mean attitude score for women with a history of natural childbirth was significantly higher than that of women with a history of CS (63 ± 9 vs 56.7 ± 9.3)(*p*<0.001) (Table 2).

**Table 2:** the Attitudes score of women with a history of previous CS vs normal delivery^a^

	Attitudes toward natural childbirth	
	Positive	Negative
	Frequency (percentage)	Frequency (percentage)
History of previous childbirth (221 individuals)		
Natural (130 individuals)	116 (89.2)	14 (10.8)
CS (91 individuals)	54 (59.3)	37 (40.6)
Total	170 (76.9)	51 (23.1)

^a^The number of women with a history of natural childbirth is 130 and CS is 91, and the percentages in this table are based on this number within the two groups with a history of natural delivery and CS

It should be noted that a woman’s attitude toward natural childbirth was not significantly correlated with occupation, but such an attitude was significantly correlated with the husband’s occupation (*p*<0.001) in a way that the occupation status of the husbands of most women with a more positive attitude to natural delivery was self-employed (20.2%) and the husband’s occupation in the majority of women with negative attitudes towards natural childbirth was employee (8.8%). Women’s attitudes were also significantly correlated with their level of education. Among the women with a positive attitude towards natural delivery, 22 and 21.2% had a diploma or high school degree, respectively (Table 3).

**Table 3:** Frequency distribution of women’s level of education based on their attitudes towards natural childbirth

	Attitude		
	Positive	Negative	
	Frequency (percentage)	Frequency (percentage)	*P*-value (One-Way ANOVA)
Pregnant women’s level of education			
Illiterate	13 (3.2)	1 (0.2)	0.02
Primary school	49 (12)	8 (2)	
High school	87 (21.2)	16 (3.9)	
Diploma	90 (22)	27 (6.6)	
Associate’s degree	25 (6.1)	14 (3.4)	
Bachelor’s degree and higher education	58 (14.1)	22 (5.4)	

A significant relation was observed between a pregnant woman’s attitude and her health insurance (*p* = 0.02): the highest scores assigned to attitudes were 64.6 ± 4.3 and 64 ± 9.7 for Rostaei insurance and military staff insurance, respectively (Table 4).

**Table 4:** Mean score for pregnant women’s attitudes towards natural childbirth based on the type of health insurance

Type of health insurance							
Mean score for pregnant women’s attitude	No insurance	Healthcare Services insurance	military staffs insurance	Rostaei insurance	Social Security insurance	Imam Khomeini Relief Committee insurance	other insurances
	51.8 ± 21.6	62.6 ± 9.9	64 ± 9.7	64.6 ± 4.3	59.5 ± 9.7	56 ± 0	60.5 ± 8.2

Acording the participants, factors affecting a positive attitude toward a natural delivery included lower maternal mortality (73%), delight to see the baby immediately after delivery (82%), establishment of a better bond between mother and baby (78%), lower incidence of infections after natural childbirth compared with CS delivery (82.4%), faster recovery (89%), faster return to daily life activities (87.6%), and lower costs of natural childbirth (85.6%). In addition, 68.2% of the women stated that if they had been aware of the complications of CS, they would not have demanded it for a previous childbirth. On the other hand, the factors affecting positive attitudes towards CS were a belief in having a healthier baby via CS delivery (56%), dislike for the position on the labor bed during vaginal delivery (55.6%), and less pain with CS compared with natural childbirth (61.4%) (Table 5).

**Table 5:** Participants attitudes toward natural childbirth and CS

List	Items	Totally agree		Agree		Disagree		Totally disagree	
		Frequency	Percentage	Frequency	Percentage	Frequency	Percentage	Frequency	Percentage
1	I like vaginal childbirth because it is a natural and acceptable method of delivery.	107	26.1	232	56.6	60	14.6	11	2.7
2	It is enjoyable for a mother to see the newborn immediately after delivery.	102	24.9	234	57.1	70	17.1	4	1
3	Emotional bonds between mother and baby are better after a natural childbirth.	106	25.9	214	52.2	80	19.5	10	2.4
4	Mothers recover as soon as possible after a natural delivery.	151	36.8	213	52	42	10.2	4	1
5	I like natural childbirth because postpartum infections in vaginal delivery are lower.	133	32.4	205	50	65	15.9	7	1.7
6	I like natural childbirth because hospital stay after a vaginal delivery is lower.	141	34.4	218	53.2	46	11.2	5	1.2
7	Return to daily life activities occurs sooner after natural childbirth.	137	33.4	219	53.4	52	12.7	2	0.5
8	Breastfeeding after natural delivery is faster and easier.	84	205	237	57.8	80	19.5	9	2.2
9	Natural childbirth costs less than CS.	142	34.6	209	51	51	12.4	8	2
10	Due to anesthesia during CS, natural childbirth is much better.	121	29.5	190	46.3	93	22.7	6	1.5
11	If I knew about the complications of CS delivery, I would never demand for it.	83	20.2	197	48	112	27.3	18	4.4
12	I prefer natural delivery because I do not like the CS scars on my abdomen.	89	21.7	208	50.7	95	23.2	18	4.4
13	Natural childbirth has lower mortality risks for mothers.	76	18.5	223	54.4	96	23.4	15	3.7
14	Babies born through CS are healthier than those born via natural delivery.	40	9.8	188	45.9	130	31.7	52	12.7
15	Possibility of infant choking, head injury and dislocated limbs in babies born via natural childbirth is higher.	15	3.7	153	37.3	190	46.3	52	12.7
16	I prefer CS because women do not like the position of the mother on the labor bed.	31	7.6	197	48	126	30.7	56	13.7
17	CS compared with natural delivery is accompanied by less pain.	67	16.3	185	45.1	108	26.3	50	12.2
18	If you wish to do tubal ligation, CS is better.	28	6.8	144	35.1	193	47.1	45	11
19	CS prevents prolapsed uterus and bladder.	14	3.4	155	37.8	173	42.2	68	16.6
20	CS prevents deformation and malformation of female genital organs.	20	4.9	149	36.3	168	41	73	17.8
21	I believe that a mother has the right to feel free to do a CS.	41	10	127	31	169	41.2	73	17.8
22	I believe that CS should be performed when it is dangerous to have natural childbirth.	11	2.7	37	9	174	42.4	188	45.9

## Discussion

The purpose of this study was to evaluate pregnant women’s attitudes towards natural and CS delivery as well as the factors affecting the delivery mode selection by pregnant women referred to healthcare centers in the city of Kermanshah in western Iran in 2016. The results of this study revealed that about 80% of the participants had a positive attitude toward natural childbirth and about 20% had a positive attitude toward CS. Although the results of the current study showed a highly positive attitude toward normal delivery, the negative attitudes are of concern. In addition, the rates in comparison with those of other studies conducted in other places are relatively high. In a similar study conducted by Pourheidari in the city of Qom in central Iran, a positive attitude toward normal delivery was reported by 94% [14]. In another investigation, 97% of pregnant women living in the city of Shahrekord (in central Iran) also had a positive attitude toward natural childbirth [15]. However, in another study in the city of Ardebil in northwest Iran, more than 70% of the pregnant women stated that they would have natural childbirth, less than in our study. The most important finding of this study was that CS was the most frequent delivery mode (59%) and that, in this respect, the medical advice by the physician was the most important factor affecting delivery mode selection [16]. The results of another study in Bushehr City (south of Iran) showed that (45.3%) of pregnant women chose normal delivery and (41.1%) chose CS. The most frequent reason for choosing CS was fear of labor pain and the most frequent reason for choosing natural delivery was fewer complications rate [17]. It seems that the reason for the difference between the attitudes of different cities of Iran toward natural delivery and CS is the different cultures of Iran, because Iran is a multicultural nation and the cultures of its various cities are different, which perhaps leads to the difference in their attitudes.

The attitude of women in western Iran toward natural delivery is at a lower level compared to other countries in the world. In a study conducted in Turkey, the majority of pregnant women selected natural delivery and less than 16% of them opted for CS. In regard to the rationale for selecting normal delivery, our results are consistent with the Turkish researchers’ findings. The most important reasons for selecting normal delivery were lower maternal mortality, delight to see the baby immediately after childbirth, better emotional bonds between mother and baby, faster recovery, faster return to daily life activities, and lower costs of this method of delivery, similar to our findings. Similarly, the most important factors affecting the selection of CS delivery included the belief in having healthier newborns, dislike of the position of women on the labor bed, less pain experienced during CS, fear of vaginal delivery, and demand for tubal litigation during the CS to prevent later pregnancy [18]. Investigating the opinions of Brazilian pregnant women, Kasai reported that the majority of women (70.8%) had considered faster postpartum recovery as the main reason for selecting natural childbirth. Pregnant women who had also chosen CS mentioned no pain during childbirth and the need for tubal ligation as the most important factors affecting the selection of this delivery mode [19]. In Lee^’^s study among South Korean women, most study participants showed more favorable attitudes toward vaginal delivery than CS. Over 95% of women preferred vaginal delivery during pregnancy and were willing to recommend this method to others [20]. Another study of Singaporean women showed that only 3.7% of them would prefer an elective CS. The most common reasons for choosing a CS were avoiding labor pains and lowering the risk of fetal distress [21].

According our findings, there was an inverse and significant correlation between an attitude toward natural delivery and a pregnant woman’s age. In this respect, Fisher argued that age can be taken into account as a factor affecting the delivery mode selection [22]. In a study by Mohammad beigi et al. in Shiraz in Iran, a significant relation was observed between maternal age and such attitudes [23]. In a similar study among pregnant women in the city of Rasht in Iran, the findings also showed no statistically significant relation between a woman’s attitude and her age [24]. Similarly, in another investigation examining pregnant women in Singapore, no significant relation was found between age and attitude [25]. In this study, the attitude was inversely correlated with the age of the pregnant woman’s husband, but such a correlation was not statistically significant. However, Ghadimi et al. reported a significant relation between the delivery mode and the husband’s age [26]. Thus, it seems that further studies should be conducted in this domain.

Likewise, the results of the present study suggested that the attitude was not significantly correlated with gestational age, which was consistent with the findings of a study conducted in Turkey [18]. The results of this study also indicated that the mean score for attitude among women with no history of delivery and those with previous delivery experience was not statistically significant, but the mean score for attitude among women with a history of natural childbirth and those with a history of CS were significantly correlated. Thus, women with a history of vaginal delivery had better attitudes towards it. In this respect, the results of a study in the city of Shahrekord in Iran showed no significant relations among the attitude of pregnant women, number of previous CSs, and delivery mode [15]. Moeini et al. also demonstrated scores for attitude towards natural childbirth higher than those of a group with elective CS and one with medical reasons, thus this value for the group with elective CS was lower than those of the other two groups [24]. Considering attitude towards delivery pain, Atghaie et al. similarly found a statistically significant difference between groups undergoing natural delivery or CS; the mean scores for a negative attitude towards natural childbirth in the group with vaginal delivery was higher than that of the group with CS [27]. A history of natural childbirth could have a positive impact on attitude towards delivery mode and its re-selection for subsequent pregnancies.

In this study, there was also a significant, positive relation between a woman’s attitude and her level of education. These results are in line with the findings of Lee, Biglari and Ghadimi [20, 26, 28]. In this study, the attitude of women towards natural delivery was not significantly correlated with occupation. These findings were consistent with the results of other studies [28, 29]. However, Mohammadi Tabar considered the source of information acquisition to be the most important factor affecting delivery mode selection and showed a significant relation between a pregnant woman’s occupation and the source of information acquisition [30]. Furthermore, a significant relation was found between a woman’s attitude towards natural delivery and her husband’s occupation, which was not in agreement with the findings by Pourheidari due to differences in the study populations [14].

In one study, 7.9% of women who chose CS stated that one of the reasons for that choice was having insurance to pay for it. Having insurance to pay for CS expenses can be related to its choice [31]. In our study a significant relation was observed between a pregnant woman’s attitude and her type of health insurance, as the highest scores assigned to attitude were for Rostaei and Military staff insurance. It seems that people with better health insurance are more concerned about their health. Generally, people who have better insurance also who work in government agencies that provide their insurance. Because of their higher level of literacy, these people are likely more aware of the disadvantages of CS and prefer natural delivery.

## Conclusion

The results of the present study suggest that most of the women studied had a positive attitude toward normal delivery as a better delivery mode for giving birth. However, far fewer felt this way than in many other nations. Given that women play a determining role in the selection of their delivery mode, training can contribute to decision-making in terms of the selection of the right one. Therefore, training and monitoring during pregnancy and giving accurate information to pregnant women is indispensible. Moreover, holding educational classes and workshops for pregnant mothers and promoting their level of awareness and attitudes considering natural delivery and CS can be useful in this respect. Furthermore, given evidence showing that a woman’s attitude is not the only important factor for selecting the normal delivery mode, more investigations seeking clarity in terms of the selected delivery method and comparing women’s attitudes with final actions, as well as the reasons behind those actions, is recommended.
